# Vibrational Energy Transfer in Organic Semiconductors Revealed by Infrared Pump–Probe Spectroscopy

**DOI:** 10.1021/acs.jpclett.6c00718

**Published:** 2026-04-24

**Authors:** Angus Currie, Jie Liu, Jack M. Woolley, James Lloyd-Hughes

**Affiliations:** † Department of Physics, 2707 University of Warwick, Gibbet Hill Road, Coventry CV4 7AL, U.K.; ‡ Department of Chemistry, University of Warwick, Gibbet Hill Road, Coventry CV4 7AL, U.K.; § X-ray Diffraction Research Technology Platform, University of Warwick, Gibbet Hill Road, Coventry CV4 7AL, U.K.; ∥ Warwick Centre for Ultrafast Spectroscopy, Research Technology Platforms, University of Warwick, Gibbet Hill Road, Coventry CV4 7AL, U.K.

## Abstract

Phonon engineering has improved charge transport in semiconducting organic molecules by introducing high mass side chains, which ameliorate harmful transient localization. The influence of these side chains on thermal energy transfer and dynamic disorder has not been fully explored. In this work, we first use low temperature X-ray diffraction to probe thermally induced structural changes in functionalized acene molecular semiconductors, combined with low temperature IR spectroscopy to track changes to their vibrational energy landscape. Furthermore, time-resolved IR pump–IR probe spectroscopy is employed to measure IR absorption kinetics associated with the high mass side chains of molecules, revealing intramolecular vibrational energy redistribution pathways. Alkyne groups in the side chains are shown to act as vibrational energy traps, remaining hot for time scales of >2 ns. The results reveal nonequilibrium vibrational pathways associated with side chains that may influence the phonon manifold relevant to dynamic disorder.

## Graphical abstract

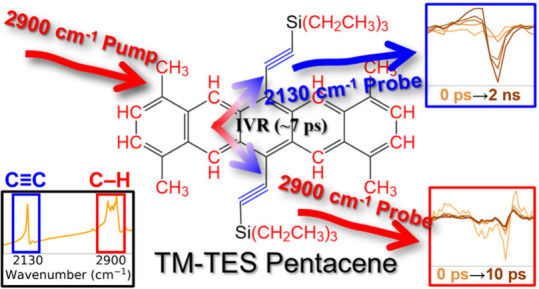

Organic semiconductors (OSCs) with novel electrical properties are of significant interest to the consumer electronics industry for their applications in thin films, including flexible and transparent displays and biodegradable electronics. Among these materials, molecular semiconductors such as pentacene have been widely studied due to their improved charge-carrier mobilities and electronic properties.^1–4^ However, charge transport in these molecules is impeded by electronic coupling with so-called “killer” phonon modes, where a small number of low-frequency optical phonons at THz frequencies can make a disproportionately large contribution to the total thermal disorder in OSC materials. This has been discussed for a range of OSCs, including pentacene, rubrene and alkyl-substituted benzothienobenzothiophene (C_*n*_BTBT).^2,5^ Recent theory has emphasized that this picture can be oversimplified: analyses restricted to isolated modes or to Γ-point phonons alone may be misleading, and broader contributions across the Brillouin zone and over a wider spectral range may also be important to describe dynamic disorder accurately.^6^

Dynamic disorder is thought to confine delocalized charge states and reduce electronic transport rates, limiting material uptake and acting as an impediment to technological development.^3,5^ However, other explanations have been proposed for charge carrier transport limits, including dipole fields in channel regions or charge trapping effects due to dielectric roughness at gate interfaces.^4,7^ The latter has been demonstrated in dibenzothiophenothienothiophene (DBTTT) devices, where as many as 95% of injected carriers are trapped due to interfacial effects.^7^

A recent rational design strategy for material development has been to use phonon engineering to reduce the impact of the THz modes.^8^ Alkan et al. found that substitution of C_*n*_BTBT molecules with long alkyl side chains (with lengths up to 12 carbons) resulted in an increase in effective mobility by up to 3 orders of magnitude, compared to the nonalkylated molecule.^9^ Schweicher et al. have examined computationally the effects of alkyl chain substitution on nonlocal Peierls-type electron–phonon coupling directly, showing corresponding fluctuations in the amplitudes of transfer integrals and site energies.^2^ However, there is limited understanding of exactly how the addition of these side chains modify the spatial distribution of delocalized charges along the π-conjugated backbone of the molecule.^10^

Time-resolved infrared (IR) spectroscopy has been employed previously in the analysis of the dynamics of thermal energy propagation in organic molecules.^11–14^ Vibrational energy flow in π-conjugated molecules in solution has been measured using time-resolved IR pump–IR probe spectroscopy by Rončević et al.^31^ In the context of molecular wires, such as ladder-type polyfluorene derivatives, current-induced heating effects are known to affect charge transport and cause chemical instability. By measuring the differential IR spectrum of tag (azide, N3) and reporter (carbonyl, CO) groups localized in different regions of substituted and unsubstituted π-conjugated fluorenyl derivative molecular wire samples, it was found that substituted side chains increased rates of *intra*- and *inter*molecular vibrational energy redistribution to hot ground reporter modes with lifetimes of around 1 ps. This is due to the increase in the vibrational density of states coupled to the excited mode, effectively providing more pathways for heat propagation and dissipation. In a similar study, Rubtsova et al.^11^ used relaxation-assisted 2DIR spectroscopy to study heat transport in oligomers (including tagged polyethylene glycol oligomers such as azPEG4) in solution for use in self-assembled monolayer junctions. Tag (azido stretch at 2100 cm^–1^) and reporter (carbonyl stretch at 1742 cm^–1^) modes were substituted at opposite ends of the molecule, and delocalized ballistic transport at rates of 11–15 Å/ps through the molecular backbone were deduced from the 2DIR spectrum, corresponding to total energy transport times around 10 ps. This work illustrated the importance of quantitatively describing the different vibrational energy transport regimes in organic materials with implications for the design of materials with controllable energy transport properties.

The above motivates a need for a deeper mechanistic understanding of the intra- and intermolecular vibrational motion of phonon-engineered OSCs with improved charge transport properties. Here, we report the use of time-resolved IR pump–IR probe spectroscopy to measure intramolecular vibrational energy transfer to reporter groups in three high mobility OSC samples with alkyne bonds: 1,4,8,11-tetramethyl-6,13-triethylsilylethynl pentacene (TM-TES, Figure 1a), 2-triethylsilyl-9,10-diphenylanthracene (TES-DPA, Figure 1b) and 2-naphthalen-2-yl anthracene (anth-napth, Figure 1c). The selective excitation of tag C—H stretching modes (2800–3100 cm^–1^, including along the aromatic backbone and within the high-mass side chains) while probing reporter alkyne C≡C stretching modes (2100–2200 cm^–1^, in the high mass side chains) provides a route to infer vibrational energy-transfer pathways and cooling time scales. The effects of temperature variation on intramolecular vibrations are explored through a combination of X-ray diffraction (XRD) and temperature-dependent Fourier transform infrared (FTIR) spectroscopy, including the role of polymorphic phase transitions and other structural disorder.^15^ The slow recovery of the C≡C stretching modes localized to the high mass side chains in TM-TES and TES-DPA demonstrates that they act as vibrational energy traps, remaining hot for time scales much longer than charge carrier or exciton lifetimes (∼50 ps^16^). Additionally, a new low temperature phase transition in TES-DPA is reported, and the potential consequences on vibrational dynamics are explored.

**Figure 1:**
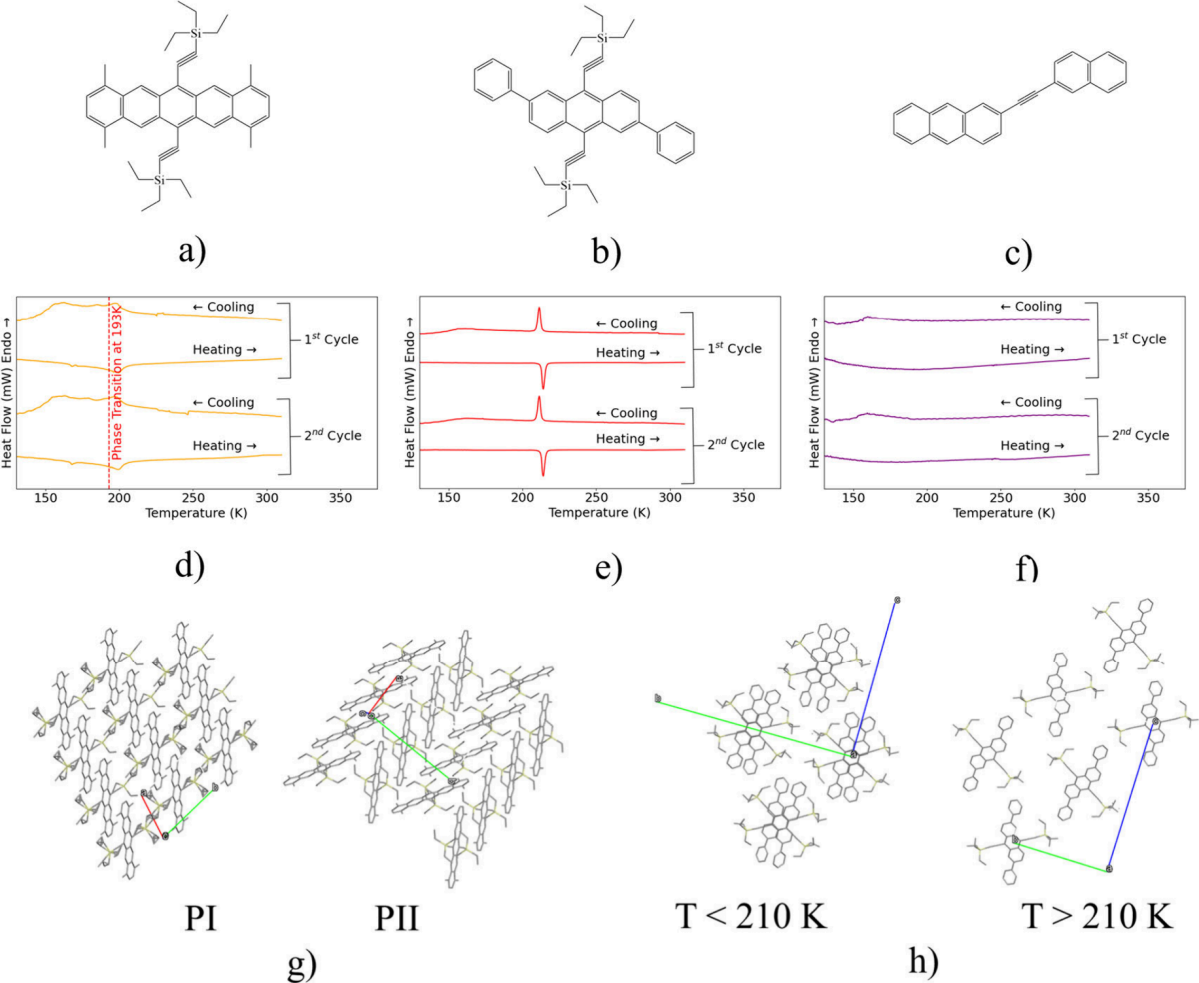
(a–c) Molecular structures of three organic semiconductor samples: TM-TES (a), TES-DPA (b), and anth-napth (c). (d–f) DSC measurements for the three samples, including TM-TES (d, orange) with highlighted phase transition at 190 K reported in the literature, TES-DPA (e, red), and anth-napth (f, purple). (g) Polymorph structures of TM-TES determined using powder XRD. PI represents a slip-stack structure present in the powder sample used in this work, and PII represents a herringbone structure exhibited by TES-DPA and anth-napth. (h) Crystal structure of TES-DPA above and below the phase transition at 210 K identified in DSC.

Powder samples provided by SmartKem Ltd. (UK) were utilized for DSC measurements and powder XRD. For the temperature dependent FTIR and IR pump–IR probe measurements, powders were processed into 13.5 mm diameter, 0.5 mm thickness KBr pellets using a high-pressure pellet press, with approximately 1 wt % molecular semiconductor. These sizes and proportions were found in preliminary studies to provide samples with an appropriate optical density for FTIR measurements. The DSC measurements were performed using a Mettler Toledo DSC 1 analyzer, with a liquid nitrogen system for a temperature range of 110–310 K. Samples of TM-TES (8.27 mg), TES-DPA (6.46 mg) and anth-napth (5.26 mg) were subjected to two consecutive temperature cycles at a rate of 10 K min^–1^ and the rate of heat flow compared to an empty (41 mg) aluminum reference pan was recorded. In the case of TM-TES, the results were compared to polymorphic transitions reported in the literature with a known detrimental effect on charge carrier mobilities.^17^ For the powder XRD structural measurements, analysis was performed on TM-TES powder using an Anton Paar XRDynamic 500 spectrometer, and structural assignment was completed using a Rietveld refinement algorithm.

The low-temperature DSC cycles are shown in Figure 1 for TM-TES (orange, Figure 1d), TES-DPA (red, Figure 1e) and anth-napth (purple, Figure 1f), with the literature reported phase transition for TM-TES at 190 K highlighted. The results show evidence of repeatable phase transitions across all three samples at 160–190 K (TM-TES), 210–215 K (TES-DPA) and 160–175 K (anth-napth). In TM-TES this confirms literature reports of a sudden shift in the length of the unit cell *b*-axis, which is associated with a negative step in stability.^18^

The results of the powder XRD analysis of TM-TES are shown in Figure 1g. Rietveld refinement analysis shows the presence of two distinct polymorphs present in the sample: a herringbone structure (labeled PI) common to many organic semiconductors and a secondary slip stack structure (PII) exhibiting modified π–π stacking. It is expected that these two polymorphs exhibit differences in electron–phonon coupling at THz frequencies due to differences in unit cell parameters and symmetries, but this has yet to be investigated. In TES-DPA, variable-temperature single crystal XRD shows that the phase transition at 210 K (Figure 1h) corresponds to a substantial change in the molecular packing within the structure. This transition likely also has a detrimental effect on charge mobility corresponding to modification of the π–π stacking structure and reduced electronic couplings characterizing hole transfers between neighboring molecules.^19^ Further research is needed to elucidate the precise nature of these transitions and their effect on the electronic properties of these materials. However, these structural changes do imply that local vibrational environments and couplings are altered with temperature, influencing the IR-active modes and the intramolecular vibrational energy redistribution (IVR) pathways tracked with pump–probe dynamics.

Infrared spectroscopy can provide further insights into how temperature changes the structure. Temperature-dependent FTIR spectroscopy was performed by using a Nicolet iS50R Research FTIR Spectrometer at a resolution of 0.5 cm^–1^, along with a Linkam FTIR600 stage and temperature control system with KBr windows. Liquid nitrogen was used as a coolant, with samples of TM-TES, TES-DPA and anth-napth cooled to 80 K, and spectra taken every 10 K up to 293 K. Room temperature FTIR measurements were separately taken using a Bruker Vertex 70 V IR spectrometer between 1000 and 3100 cm^–1^ with a resolution of 0.1 cm^–1^. Temperature dependent FTIR has been shown to be a sensitive probe of vibrational anomalies, including charge transfer states in organic semiconductors.^20^

Figure 2 shows the absorbances from temperature-dependent FTIR spectroscopy on the three samples. From the room-temperature survey spectra in Figure 2a, we focus on two regions of interest: the alkyne stretch modes at 2100–2200 cm^–1^ and the C—H stretch modes from 2900 to 3100 cm^–1^. The C—H stretch modes were assigned using standard IR-activity group/wavenumber correlations:^21^ the sharper peaks around 3050 cm^–1^ correspond to aromatic sp^2^ C—H stretches, while alkyl sp^3^ C—H stretches occur just below 3000 cm^–1^. Anth-napth has the strongest contribution to the absorbance from aromatic stretches, and with no alkyl groups, the peaks around 2950 cm^–1^ seen in TM-TES and TES-DPA are absent. There are multiple different alkyl environments in TM-TES, with ethyl groups in the TES side groups as well as methyl groups connected to the backbone (Figure 1). In TES-DPA there are still alkyl groups on the two TES side chains, but there are no backbone methyl groups.

**Figure 2:**
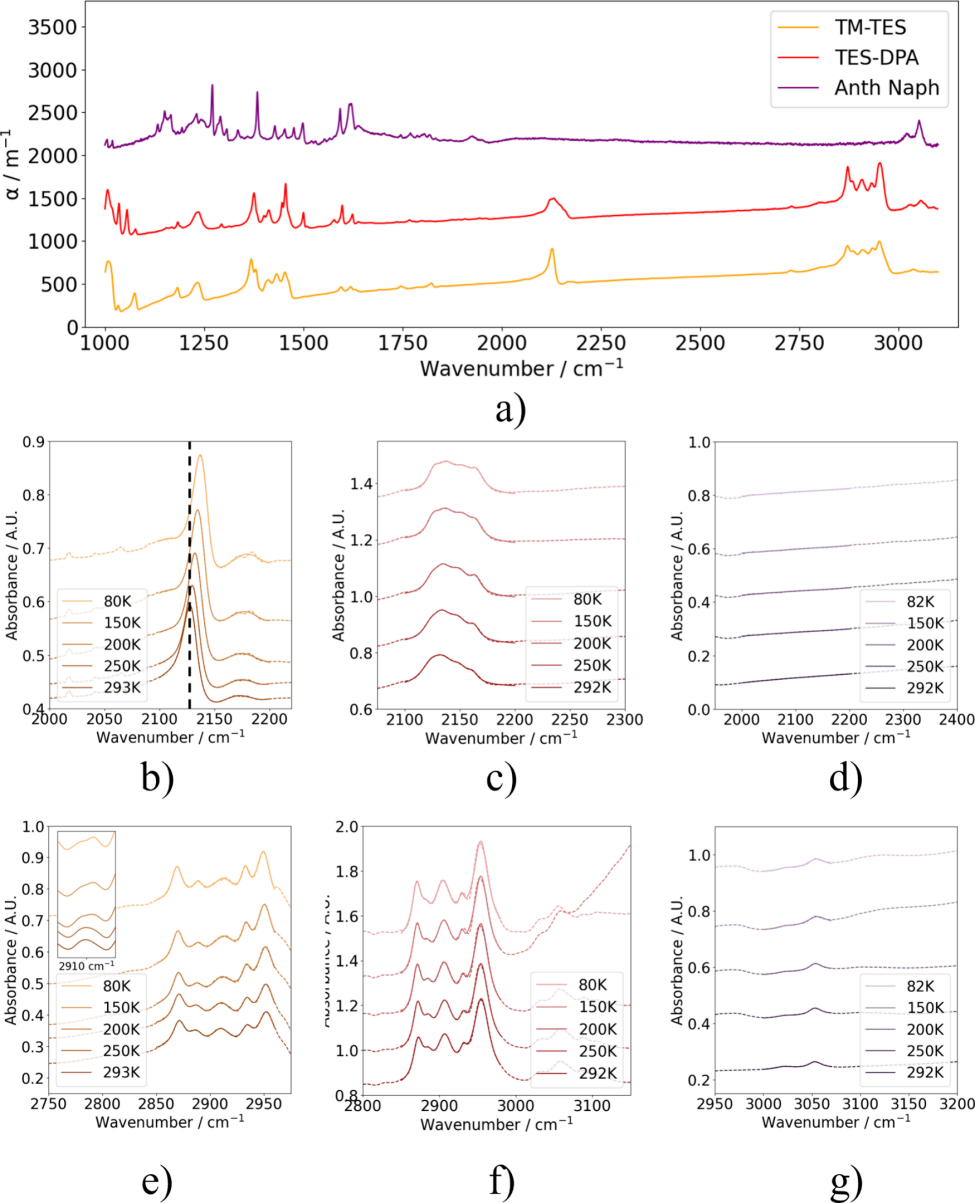
(a) Room temperature FTIR spectra (vertically offset) for three organic semiconductor samples (TM-TES, orange; TES-DPA, red; anth-napth, purple) in the region 1000–3100 cm^–1^, showing fingerprint region, alkyne stretch modes in TM-TES and TES-DPA at around 2130 cm^–1^, and C—H stretch modes at around 2900–3000 cm^–1^ in all three samples. (b–g) FTIR temperature dependence in the alkyne stretch region (b–d) and C—H stretch region (e–g), from 80 K (light) to 293 K (dark) in all three samples. Raw data are shown by the dashed line, and fitted data by the solid line. Panels (b) and (e) show TM-TES, panels (c) and (f) show TES-DPA and panels (d) and (g) anth-napth.

Temperature-dependent FTIR absorbance data reveal subtle shifts in the alkyne, alkyl, and aromatic vibrational modes with temperature, as shown in Figure 2b–g. The raw data are shown with dashed lines, while the solid lines represent fits to the data via a sum of Lorentzian functions representing the absorption peaks and a linear scatter term, from which changes in peak parameters with temperature were modeled (further details are given in the Supporting Information). All samples showed peak sharpening at low temperatures due to reduced thermal disorder affecting phonon scattering processes.^22^ Temperature-induced wavenumber shifts are also observed in several modes, as thermal expansion alters bond length and strengths. With increased temperature, the alkyl modes at 2950 cm^–1^ blue shift while the alkyne modes at 2130 cm^–1^ red shift in both TM-TES and TES-DPA. Changes at phase transition temperatures in these samples include peak splitting in TM-TES at 2910 cm^–1^ across the 193 K phase transition (inset in Figure 2e), and the emergence of new modes at 210 K in TES-DPA at 2147, 2162, and 2884 cm^–1^.

The alkyne stretch range for each sample is significantly different: as evident in Figure 2b–d, TM-TES has a single prominent mode, TES-DPA has 4 modes, and anth-napth does not have any IR absorption features at 2100–2200 cm^–1^. We attribute the extra modes for TES-DPA to multiple distinct local bond environments for the side chains owing to its different crystal structure (Figure 1): the higher temperature phase has fewer unique bond environments and 2–3 distinct IR modes, compared to the lower temperature phase in TES-DPA (4 distinct IR modes). Anth-napth has similar molecular structure on either side of the alkyne bond, reducing its dipole moment and thereby preventing vibrational absorption according to standard IR selection rules.^23^ The results in Figure 2d show that this symmetry is not lifted at temperatures as low as 80 K. For this reason, anth-naphth was not used for the transient vibrational absorption analysis.

IR pump–IR probe spectroscopy was used to study the dynamics of thermal energy transfer, corresponding to intramolecular vibrations localized in different parts of the molecule. For both degenerate and nondegenerate pump–probe experiments, femtosecond mid-IR pulses centered on the energies corresponding to key vibrational features in the molecule were generated from two optical parametric amplifiers (TOPAS, Light Conversion) pumped by a Ti:sapphire laser amplifier (Newport Spectra Physics Spitfire ACE). The IR pulse length, which corresponds to the experimental time resolution, was approximately 100 fs. The pump–probe time delay was controlled by using a mechanical delay stage with a maximum time delay of 2 ns. The pump and probe pulses were cross-polarized by using an optical periscope system, and the pump pulse scatter from the KBr pellet sample was eliminated by using a pair of wire grid polarizers. The probe pulse was detected using a Mercury Cadmium Telluride detector array, and a differential chopping scheme was employed to measure the transient vibrational absorption spectra at a resolution of 3.4 cm^–1^ after calibration. The pump pulse (spectral coverage from 2750–3100 cm^–1^) photoexcited the tag modes: C—H stretches that are present in the conjugated backbone of the three samples, as well as within the side chains of the molecule, while the probe sampled either the alkyl range below 3000 cm^–1^ or the alkyne stretches (2000–2250 cm^–1^). Transient kinetics were modeled using a modified exponential expression including IRF contributions.^24^

Figure 3 shows the results of degenerate (same color) pump–probe spectroscopy of the C—H stretches of TM-TES and TES-DPA, including the differential absorbance spectra, ΔOD, between 0 and 10 ps (Figure 3a,b). The C—H range includes five distinct absorption modes in FTIR in both samples that overlap significantly, meaning the spectra in Figure 3a,b exhibit a combination of multiple ground state bleaches (negative ΔOD) and excited state absorption-like features (positive ΔOD) from these modes. For TM-TES, with a single alkyne stretch mode, the transient absorption has substantial positive ΔOD at 2971 cm^–1^ that is likely a result of a vibrational blue shift of the mode at 2960 cm^–1^ as the bond heats up locally, following the trend shown in the low temperature FTIR in Figure 2e. This can be regarded as a hot ground state, where the energy injected into the molecule by the pump alters the resonance seen by the probe pulse.^25,26^

**Figure 3:**
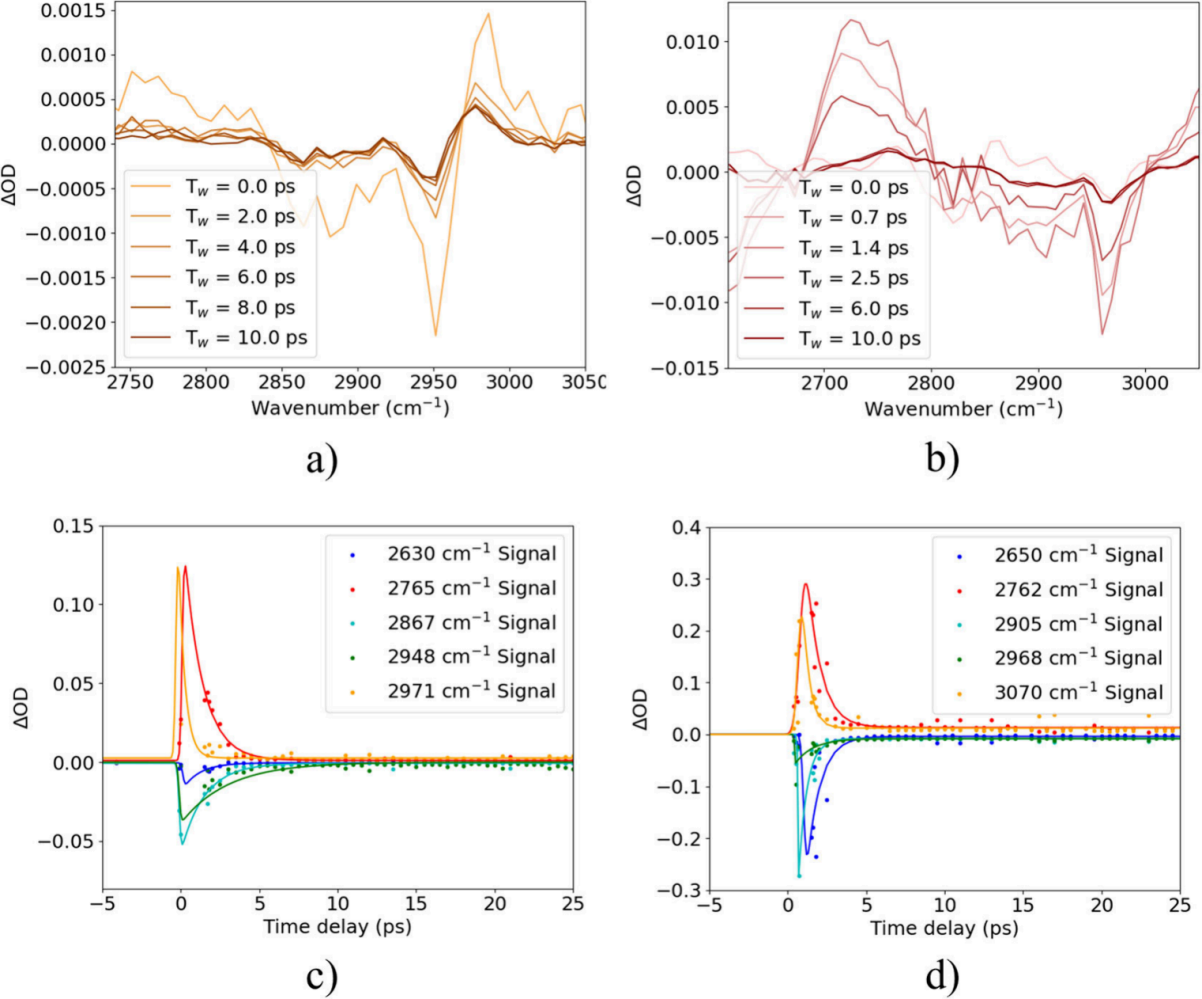
(a,b) Degenerate (2900 cm^–1^ pump–2900 cm^–1^ probe) transient vibrational absorption spectra for TM-TES (a) and TES-DPA (b), up to a probe delay of 10 ps. (c,d) Ultrafast dynamics of features corresponding to GSB (blue, cyan, green) and ESA (red, orange) peaks in the differential spectra, up to a probe delay of 25 ps.

To track the vibrational population dynamics of these modes, Gaussian fits to the positive and negative ΔOD spectral features were made at each time delay, at center frequencies labeled in the captions, before integrating to give the transients reported in Figure 3c,d. The lifetimes of the five transient peaks are all between 1 and 3 ps for both TM-TES and TES-DPA, showing that alkyl modes in these molecules relax quickly through intramolecular vibrational energy redistribution to other sites. These are typical lifetimes for π-conjugated molecules, including molecular wires and oligomers.^11,12^ Degenerate IR pump–IR probe spectroscopy of the alkyl stretch modes in anth-naphth at 3000 cm^–1^ was also attempted, but no transient signal was detected due to increased optical scattering in this sample (see Supporting Information).

To examine energy transfer to the alkyne mode, we report in Figure 4 the results of nondegenerate 2900 cm^–1^ pump–2100 cm^–1^ probe of TM-TES and TES-DPA, where energy is injected into the C—H bonds throughout the molecule and the response of the C≡C stretch modes linking to the high mass side chains was recorded. Here the excited state absorption/ground state bleach-like transient signal can be observed to increase in magnitude with pump–probe delay (Figure 4a,b in color). The spectral shape of the signal is similar to the FTIR difference spectra with temperature (black lines), and hence the transient spectra can be explained as corresponding to a hot ground state. The energy differences of the positive and negative peaks in Figure 4a,b are 11.5 ± 1.6 cm^–1^ (1.42 ± 0.20 meV) and 35.8 ± 4.7 cm^–1^ (4.44 ± 0.58 meV) for TM-TES and TES-DPA, respectively, at late times (>20 ps) but are significantly reduced at early times (<10 ps). This is due to the wavenumber of the peak red-shifting with increasing temperature, as shown in Figure 2b,c. The dynamics of this thermal red shift are shown in Figure S7c,d of the Supporting Information, where further discussion on the assignment of this signal can also be found.

**Figure 4:**
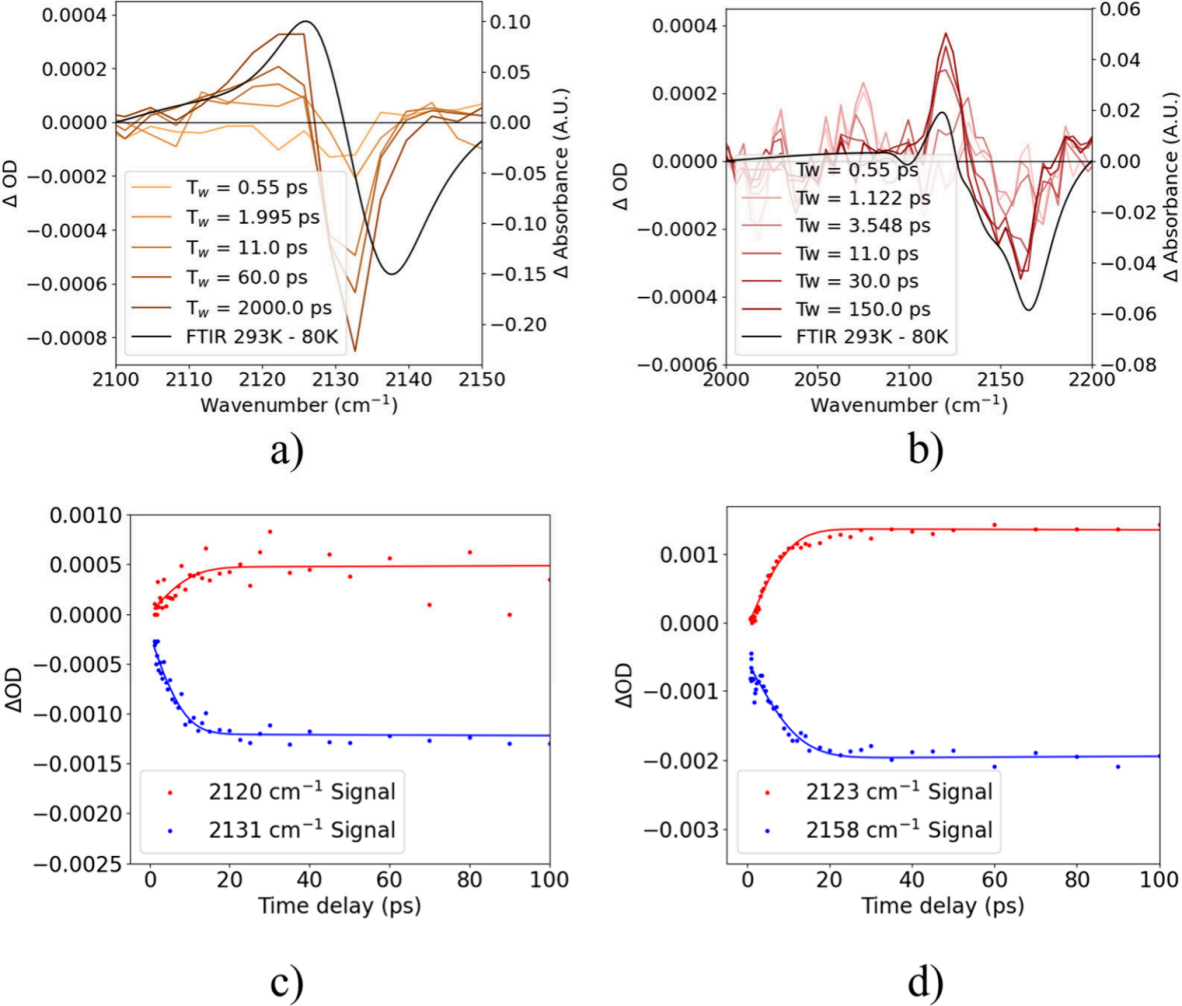
(a,b) Nondegenerate (2900 cm^–1^ pump–2130 cm^–1^ probe) transient vibrational absorption spectra for TM-TES (a), up to a probe delay of 2 ns, and TES-DPA (b), up to a probe delay of 150 ps. For both samples, the differential temperature dependent FTIR spectrum is shown in black between 293 and 80 K for comparison. (c,d) Ultrafast dynamics of features corresponding to GSB (blue) and ESA (red) peaks in the differential spectra, up to a probe delay of 100 ps.

The dynamics of the features (Figure 4c,d) show an exponential rise with time constants of 7.69 (±0.41) and 9.24 (±0.67) ps for the positive and negative peaks respectively in TM-TES, and 6.5 (±0.45) and 8.4 (±0.55) ps for the positive and negative peaks respectively in TES-DPA. These time constants are significantly longer than the <3 ps decay of the C—H stretch modes. The slower signal kinetics suggest that IVR likely occurs through a series of intermediate states, where vibrational energy travels to different bonds before exciting the C≡C stretching mode. Most likely, these intermediate states are C—C and C=C stretch bonds in the conjugated region of the molecule, although further work is required to confirm this. Also of note is that TES-DPA exhibits somewhat faster nondegenerate signal growth than TM-TES, which may be linked to differences in backbone rigidity, local side-chain environments, or both; the data presented here do not distinguish the origin of this effect.

Based on average room temperature interbond distances from backbone C—H groups to side chain C≡C bonds of 9–12 Å measured in single crystal XRD, the vibrational energy transfer rate is estimated to be 1–2 Å/ps, based on the signal growth rates of 6–9 ps. This is consistent with the high scatter, downhill dissipative heat transport in oligomers. This estimate is approximate and assumes averaged uniform vibrational energy transfer from several excited alkyl sites through intermediate alkyl and alkene bonds in the molecular backbone. It also assumes minimal molecular deformation from the room temperature structure when the molecule is excited, which may alter the interbond distances.

In the nondegenerate pump–probe experiment, the differential absorption persists for much longer times without decaying, in excess of 2 ns (the maximum waiting time of the experimental setup) across both samples (Figure 4c,d). This is evidence that the side-chain alkyne stretching coordinate can host a long-lived hot-ground-state population, whereby thermal energy cannot rapidly escape to other molecular modes or the phonon bath due to weak anharmonic coupling. This is an unusually long relaxation time for a transient vibrational absorption signal. However, lifetimes of a similar range have been observed by Cho et al., where hot ground state signals due to photothermal relaxation of molecules in solution were estimated to be 1–10 ns.^25^

We now discuss the comparison of the transient spectra and the temperature difference spectra in more detail, as shown in Figure 4a,b where the differential FTIR spectrum between 293 and 80 K is shown in black for both samples, with a strong similarity to the late time pump–probe transient signal. This is indicative that the differential spectra are consistent with enhanced lattice temperatures, as the transient data were obtained at room temperature (not 80 K). Extrapolating the temperature-dependent FTIR absorption upward from room temperature (Supporting Information) would lead to the observation that the transient spectra are consistent with temperatures of the probed vibrational coordinates of about 450 K (TM-TES) and 600 K (TES-DPA). A true bulk temperature rise of several hundred kelvin would be expected to cause irreversible structural or chemical changes in the samples, which was not observed. We therefore interpret the spectroscopically inferred 450–600 K values as effective temperatures of the excited vibrational coordinate, rather than literal equilibrium crystal temperatures. Further, the temperature of the crystal is unlikely to have risen by ≫100 K after the absorption of the pump beam: an order-of-magnitude estimate suggests the overall temperature increase of the excited sample region is <2 K per pulse. This is based on values for pump excitation area ≈1 mm^2^, penetration depth ≈100 μm, density ≈1500 kg m^–3^, pump laser pulse energy 760 μJ, the fraction of the broadband pump absorbed (≈20%) and the measured heat capacity from DSC (≈500 J kg^–1^ K^–1^). The transient spectra therefore show that local hot ground state effects in the nonequilibrium case raise the effective temperature of the modes to much higher temperatures than if the entire lattice was warmed by the absorbed pump laser pulse energy.

The distinction between the two methods of heating is that an ultrafast pump creates a nonthermal, mode-specific vibrational population, whereas heating the crystal creates a thermalized distribution. Thus, the present experiment probes how selective population of high-frequency intramolecular coordinates may couple into, or temporarily bottleneck, the wider vibrational manifold relevant to dynamic disorder,^6^ rather than directly identifying a single transport-limiting phonon.

Thermal energy traps may have implications for charge dynamics. To summarize the time scales uncovered: the initial IVR within the excited C—H stretching manifold takes <3 ps, while energy transfers to the alkyne linker within 6–9 ps. The alkyne linker remains hot for >2 ns, suggesting that thermal transport away from this mode is very slow. To quantify the implications of this trapping on disorder, we treat the C≡C stretch approximately as a localized diatomic stretch coordinate (*ṽ*_0_ = 2130 cm^–1^, μ = 6 amu). The zero point RMS displacement for this system is $\frac{\hbar }{2\mu \omega_{0}}$ = 0.0363 Å. At temperature *T* = 293 K the quantum RMS coordinate fluctuation ⟨*q* ^2^⟩ is^28^

1 $$\left\langle q^{2}\right\rangle =\frac{\hbar }{2\mu \omega_{0}}\;\coth\left(\frac{\hbar \omega_{0}}{2k_{B}T}\right)\approx 0.03633\;Å$$

Within this simple local-mode estimate, the small difference between root mean square (RMS) displacement at room temperature and zero temperature implies that, at room temperature, the alkyne bond displacement is dominated by zero-point motion. Even if the effective mode temperatures were as high as inferred from thermal-red shift mapping from FTIR (450–600 K), the additional thermal RMS contribution to the bond displacement would be only 0.002–0.004 Å. The small thermal increment suggests that trapped energy in the alkyne coordinate is unlikely, by itself, to generate large structural distortions that directly control charge transfer integrals. A more plausible picture is that these high-frequency vibrations perturb vibrational relaxation pathways and anharmonic coupling into the broader phonon manifold, including the lower-frequency intermolecular motions most closely associated with dynamic disorder. Alternatively, charge transport may be impacted positively through vibrational bottleneck effects: by acting as long-lived sinks for vibrational energy, these alkyne groups may impede the dissipation of heat from the lattice. While these side chains are designed to engineer phonons and suppress transient localization, we suggest that their vibrational energy relaxation introduces an additional, nonequilibrium consideration for high-performance organic semiconductors.

In conclusion, we present results confirming temperature-induced structural and dynamical changes in a range of functionalized acene molecular semiconductors, principally, TM-TES and TES-DPA. A newly identified low-temperature phase transition in TES-DPA is shown and discussed in the context of charge dynamics and effects on vibrational behavior as measured by low-temperature FTIR. In a third OSC, anth-napth, no significant temperature dependent vibrational changes—such as new peaks due to symmetry breaking or phase transitions—were observed in the C—H or C≡C stretch ranges.

Transient IR absorption spectroscopy was used to establish pathways of heat transfer in TM-TES and TES-DPA. When vibrational energy was injected into the C—H stretching tag modes, dissipative heat transfer was able to redistribute thermal energy to the rest of the molecule in 1–3 ps. Within 6–9 ps, vibrational energy was transferred to the side-chain alkyne stretch coordinates, at an estimated rate of 1–2 Å/ps. Finally, alkyne modes in the high mass side chains entered a long-lived hot ground state where thermal energy was trapped for time scales longer than 2 ns. This lifetime is significantly longer than those of excitons in similar molecules, which are typically on the order of 50 ps. Excitation of the alkyne modes may modulate thermal relaxation pathways, thereby potentially modifying the disordered, low-frequency phonons in the crystalline lattice.

Experimental limitations include the overlap of C—H stretch modes in the tag energy range, causing uncertainty in the exact pathways of the vibrational energy flow. Further coherent effects like PFID,^29^ or additional pump scatter near zero pump–probe delay, make short-lived excited state signals challenging to measure. A complementary study of “uphill” thermal energy transport could help quantify heat propagation rates and pathways as well as the study of other potential reporter features such as C=C stretches in the charge-transport active backbone of the samples. Extending this investigation to low temperature IR pump–IR probe spectroscopy could reduce the effects of thermal diffusion to better distinguish vibrational energy trapping from pump laser-induced heating. Additionally, measuring the fluence dependence of the transient signal would help disentangle the dynamics of single- and multiphonon vibrational energy flow.^30^ To reduce scatter, particularly at low pump powers, thin film samples of OSCs can also be considered. Future work may deploy other techniques such as 2D-IR, visible pump–IR probe, or computational modeling to explore these interactions more fully, including directly measuring intramolecular coupling and any effects on delocalized electronic states.

Finally, the present results suggest that, in addition to equilibrium phonon-engineering, nonequilibrium vibrational energy redistribution into the broader phonon manifold may also be relevant to dynamic disorder.

## Supplementary Material



## Notes

Powder X-ray diffraction data and crystallographic data; temperature-dependent FTIR spectra and fits; lattice parameters; additional pump–probe spectra and calibration spectra (PDF)
