# Supplementary material for: Vibrational Energy Transfer in Organic Semiconductors Revealed by Infrared Pump–Probe Spectroscopy
Source: J Phys Chem Lett. 2026 Apr 24;17(18):5249–57. doi: 10.1021/acs.jpclett.6c00718 (PMC13158985; doi:10.1021/acs.jpclett.6c00718)
Supplement: Supplementary file 1 [file jz6c00718_si_001.pdf]

Supplementary Information for:
Vibrational Energy Transfer in Organic Semiconductors Revealed by Infrared Pump-Probe Spectroscopy

Angus Currie[1,2], Jie Liu[1,3], Jack M. Woolley[1,4], James Lloyd-Hughes[1,4,*]

1. University of Warwick, Department of Physics, Gibbet Hill Road, Coventry, CV4 7AL, UK.
2. University of Warwick, Department of Chemistry, Gibbet Hill Road, Coventry, CV4 7AL, UK.
3. X-ray Diffraction Research Technology Platform, University of Warwick, Gibbet Hill Road, Coventry, CV4 7AL, UK.
4. Warwick Centre for Ultrafast Spectroscopy, Research Technology Platforms, University of Warwick, Gibbet Hill Road, Coventry, CV4 7AL, UK.

* Corresponding author. Email: j.lloyd-hughes@warwick.ac.uk

Powder X-Ray Diffraction of TM-TES and Low Temperature Single Crystal X-Ray Diffractometry of TM-TES and TES-DPA

Powder XRD was used to study the crystal structures of TM-TES powder samples at room temperature. Results are shown in Figure S1. Analysis revealed two polymorphs, Form I and Form II as described previously in the literature(1). The temperature dependence of Form I was subsequently studied between room temperature and 150 K, covering the range of the phase transition identified in DSC. The variation of the unit cell parameters with temperature is shown in Figure S1 and in Table S1.

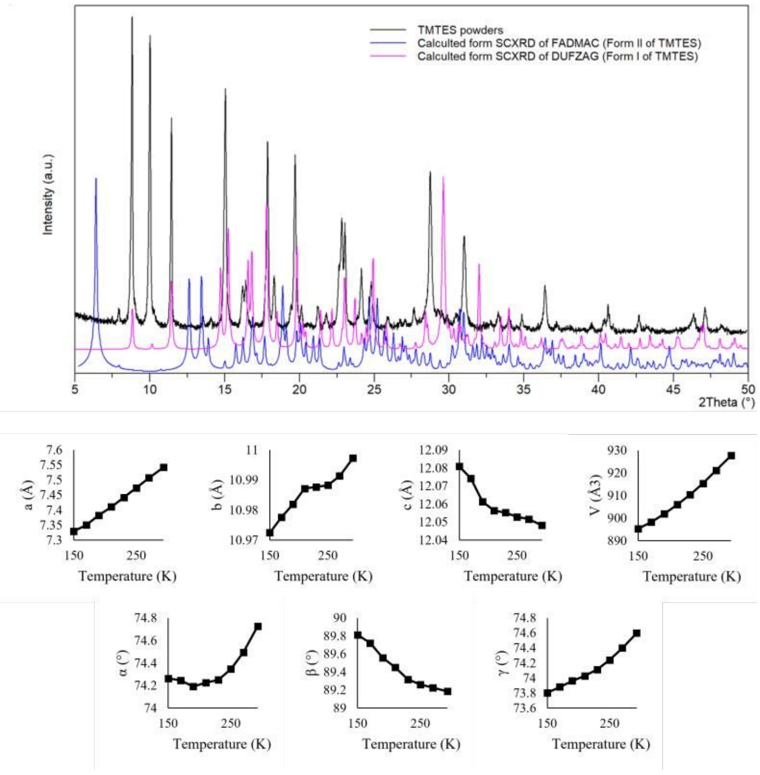

Figure S1) Upper: Results of powder XRD at room temperature of TM-TES, and the calculated spectra for polymorphs designated Form I and Form II in the 2θ range of 5–50°. Lower: temperature dependence of unit cell parameters of TM-TES during cooling from 293–150 K, showing the significant change in the b-axis length at 193 K.

Single crystals of TES-DPA were also analysed using XRD at low temperatures (293–100 K), revealing details of a phase transition found in DSC at 210 K. This transition results in the doubling of the *b*-axis from 17 to 34 Å and is associated with significant changes in the FTIR spectrum (including peak splitting and sharpening) due to altered molecular environments(2), which can be seen in figure 2 of the work. The lattice parameters for this analysis are shown in figure S2 and table S2. Further research (such as low temperature IR pump–IR probe) is needed to explore whether these new molecular environments modify vibrational energy redistribution pathways and charge formation pathways due to altered π-π stacking in this material.

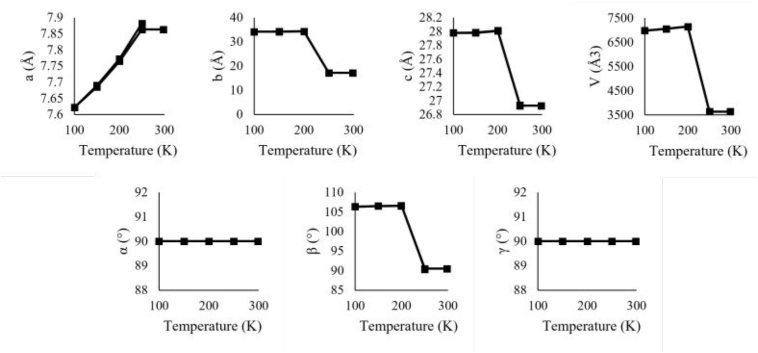

Figure S2) Temperature dependence of lattice parameters in TES-DPA during cooling and heating from 293–100 K, showing phase transition at 210 K including doubling of the b-axis length as two molecules in the unit cell become non-equivalent.

Temperature (K)		a / Å	b / Å	c / Å	α / °	β / °	γ / °	V / Å³
Cycle 1 – cooling	293	7.5425	10.9973	12.0482	74.727	89.186	74.600	927.78
	270	7.5073	10.9914	12.0517	74.496	89.227	74.401	921.15
	250	7.4741	10.9883	12.0529	74.347	89.263	74.241	915.39
	230	7.4420	10.9877	12.0553	74.251	89.318	74.114	910.45
	210	7.4107	10.9872	12.0564	74.227	89.452	74.028	905.97
	190	7.3829	10.9820	12.0614	74.191	89.560	73.964	901.91
	170	7.3506	10.9776	12.0742	74.246	89.721	73.882	898.21
	150	7.3298	10.9725	12.0809	74.264	89.813	73.805	895.34
Cycle 2 – heating	170	7.3581	10.9781	12.0671	74.226	89.702	73.902	898.67
	190	7.3923	10.9787	12.0498	74.181	89.560	73.991	902.00
	210	7.4269	10.9787	12.0403	74.140	89.278	74.073	906.05
	230	7.4677	10.9742	12.0330	74.194	89.225	74.182	910.95
	250	7.5083	10.9712	12.0253	74.300	89.173	74.324	916.31
	270	7.5490	10.9710	12.0210	74.454	89.128	74.492	922.53
	290	7.5875	10.9750	12.0153	74.692	89.121	74.709	929.30

Table S1) Lattice parameters for low temperature single crystal XRD of TM-TES Form I, as shown in figure S1.

Temperature (K)		a / Å	b / Å	c / Å	α / °	β / °	γ / °	V / Å³
Cycle 1 – cooling	298	7.8632	17.1913	26.9265	90	90.491	90	3639.8
	250	7.8632	17.1913	26.9265	90	90.491	90	3639.8
	200	7.7647	34.2777	28.0108	90	106.559	90	7146.1
	150	7.6852	34.1935	27.9840	90	106.489	90	7051.4
	100	7.6216	34.1224	27.9795	90	106.332	90	6982.9
Cycle 2 – heating	150	7.6895	34.1867	27.9791	90	106.498	90	7052.3
	200	7.7723	34.2611	28.0060	90	106.576	90	7147.7
	250	7.8819	17.1464	26.9346	90	90.347	90	3640.0
	298	7.9889	17.1034	27.0740	90	90.493	90	3699.2

Table S2) Lattice parameters for low temperature single crystal XRD of TES-DPA, as shown in figure S2.

Low temperature FTIR of TM-TES and TES-DPA

TM-TES and TES-DPA were both analysed using temperature dependent FTIR in the range 80–293 K so as to measure changes in the vibrational characteristics of the two samples due to thermal effects. The raw data was fit to a linear sum of contributions from Lorentzian functions corresponding to absorption features and a linear scatter term. In TES-DPA , this feature is represented by four overlapping Lorentzian functions due to the different environments of the alkyne bonds in across the two non-equivalent molecules in the unit cell. As the samples heat up, the alkyne stretch features at 2130 cm^{-1} broaden and shift to lower wavenumbers, and so result of taking a differential spectrum between two temperatures is a signal that looks superficially similar to the ground state bleach (GSB) and excited state absorption (ESA) signal of an IR pump–IR probe spectrum of a three-level anharmonic system, with an energy difference between the positive and negative peaks as a measure of the thermal redshift. This is shown for both samples in figure S3. The thermal redshift values are similar to energy differences in the nondegenerate IR pump–IR probe spectra in figure 4 of the work, and so it can be inferred that the experimental results are due to laser induced heating creating a hot ground state, rather than an actual vibrational excitation due to coupling to the excited tag modes elsewhere in the molecule(3).

The temperature dependence of IR scattering in these samples is influenced by the anharmonic effects of optical phonons, as described by Balkanski *et al.*(4). Three- or four-phonon processes corresponding to cubic and quartic anharmonic terms respectively affect trends in the linewidth *Γ(T)* and frequency shifts *Δ(T)* with changing temperature according to equations S1 and S2:

$$\Gamma(T) = A\left(1 + \frac{2}{e^{x}-1}\right) + B\left(1 + \frac{3}{e^{y}-1} + \frac{3}{(e^{y}-1)^{2}}\right) \quad \text{(S1)}$$

$$\Delta(T) = C\left(1 + \frac{2}{e^{x}-1}\right) + D\left(1 + \frac{3}{e^{y}-1} + \frac{3}{(e^{y}-1)^{2}}\right) \quad \text{(S2)}$$

where

$$x = \frac{\hbar\omega_0}{2k_BT}, \qquad y = \frac{\hbar\omega_0}{3k_BT}$$

In this context, the *A* and *C* terms describe the cubic contribution to anharmonicity, while the *B* and *D* terms describe the quartic contribution. By extracting the changes in linewidth and peak wavenumber with temperature from the fitted data, it is possible to extract these terms and so determine the contributions to changes in the IR absorption spectra for the key features in the study. This is shown for the case of TM-TES, which has only a single alkyne stretch mode at 2130 cm^{-1} which simplifies the modelling. However, TES-DPA as several overlapping stretch modes in this region, and the fitting parameters for some of these have sudden jumps at 210 K due to the phase transition discussed above. For this region, temperature dependent changes in the fitting parameters for TES-DPA are modelled linearly above 210 K. The results of this for both samples are shown in figure S4.

Note that the above model has been derived for the case of the temperature dependence of Raman scattering by optical phonons in crystalline silicon, rather than for trends in the absorption profiles of covalent bonds in organic molecules. In this case, absorption may be affected by other physical processes which are not considered by the model, such as intramolecular coupling. The model has been chosen here because vibrational relaxation in both systems is governed by thermal changes in anharmonicity and multi-phonon (or multi-mode) decay pathways to lower energy modes, and so these equations are expected to reflect variance in the data due to these trends.

Finally, using the modelled fitting parameters described above, it is possible to extrapolate the differential FTIR spectra of these two molecular semiconductors at higher temperatures compared to 293 K, by substituting higher values of *T* into equations S1 and S2, and the linear models for TES-DPA, rather than by taking new spectra at higher temperatures experimentally. From this, continued trends in thermal redshift can also be estimated. These results are shown in figure S5. The energy difference between peaks seems to increase approximately linearly with temperature as the absorption features continue to redshift. For these samples, the thermal redshift reaches the values measured in IR pump–IR probe at approximately 450 K for TM-TES (1.42(±0.20) meV*)* and above 600 K for TES-DPA (4.44 (±0.58) meV). However, this does not imply that the samples are reaching these high equilibrium temperatures in the transient absorption experiment; as discussed in the work, the total local heating due to the laser is only around 0.1 K per pulse. The extrapolated trends in differential spectra serve to demonstrate that local heating of the alkyne stretch mode above room temperature is able to produce similar thermal redshifts in differential spectra to the nonequilibrium pump–probe measurements, further suggesting that the transient signals in figure 4 of the work are due to hot ground state effects across both samples.

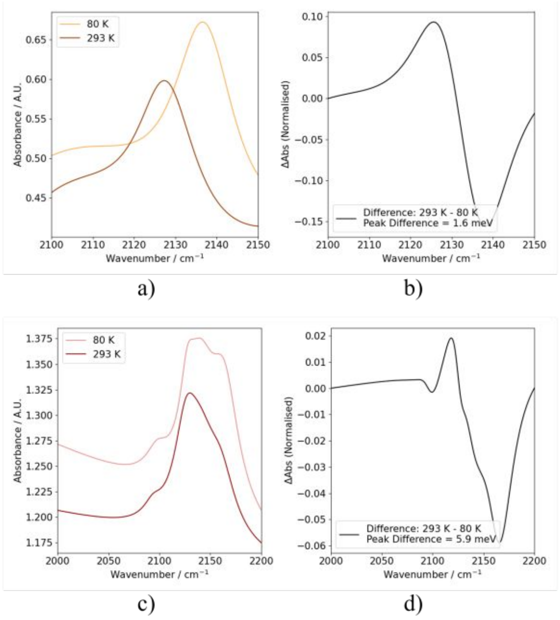

Figure S3) (a) FTIR spectrum of TM-TES showing alkyne stretch mode at 2130 cm^{-1} at 80 and 293 K, (b) difference spectrum of the FTIR spectra of TM-TES between 293 (hot) and 80 (cold) K with the energy difference between the positive and negative peaks highlighted in the legend, visually very similar to the nondegenerate IR pump–IR probe spectrum of TM-TES in figure 4a of the work and with a similar energy difference: 1.6meV compared to 1.42(±0.20) meV. (c) FTIR spectrum of TES-DPA showing alkyne stretch mode at 2130 cm^{-}1 at 80 and 293 K, and (d) difference spectrum between these two temperatures with peak energy difference similar to the nondegenerate IR pump–IR probe spectrum of TES-DPA in figure 4b of the work: 5.9 meV compared to 4.44 (±0.58) meV.

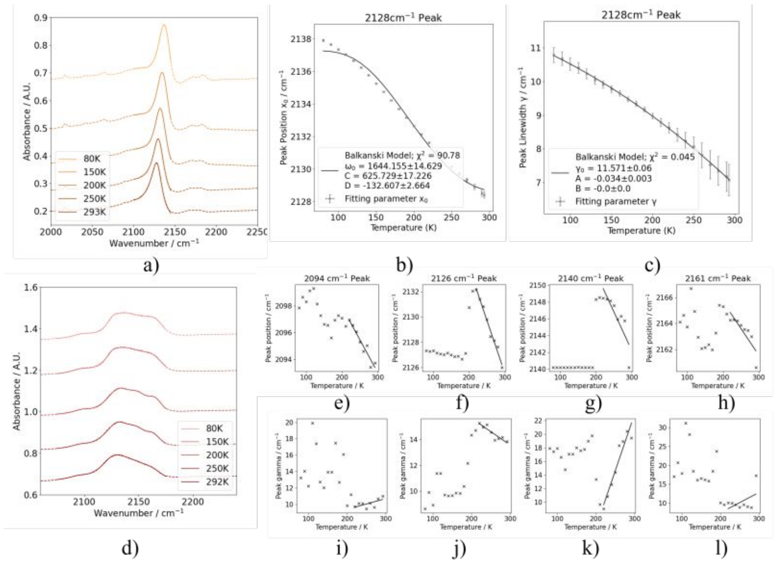

Figure S4) (a) Temperature dependent FTIR spectrum of TM-TES at 2000–2250 cm^{-1} between 80 and 293 K, raw data (dashed line, orange) and data fitted via the sum of a Lorentzian function and a linear scatter term (solid line, orange). The temperature dependence of the Lorentzian model is shown for (b) the peak wavenumber ω_0 and (c) the linewidth γ. The solid lines in (b) and (c) are the fitted temperature dependence model (equations S1 and S2), with the values for A, B, C and D shown in the figure legend, representing the contributions of three- and four- phonon optical scattering processes. (d) shows the temperature dependent FTIR spectrum of TES-DPA over the same range, with the raw data (dashed line, red) fitted to a sum of four Lorentzian functions centred on 2094, 2126, 2140 and 2161 cm^{-1} and a linear scatter term (solid line, red). (e-h) show the temperature dependence of the central wavenumbers ω_0 of these peaks, and figures (i-l) show the temperature dependence of the linewidths γ of these peaks. Due to the phase change in TES-DPA at 210 K, several of these values show sudden jumps around this temperature. Therefore, rather than use the model from equations S1 and S2, a linear fit for the range of 210–293 K is shown by the solid lines in these plots.

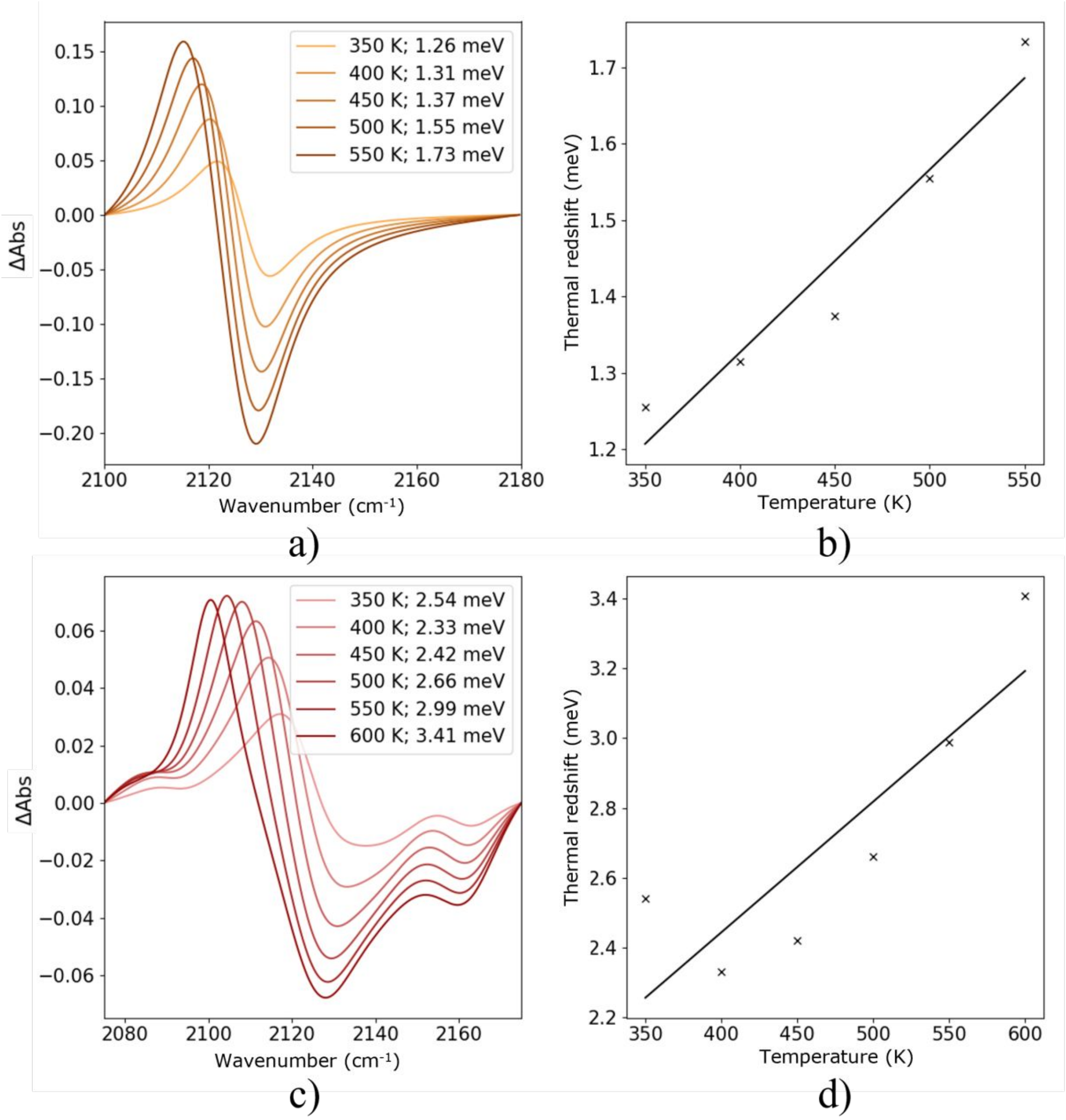

Figure S5) Results of extrapolating the trends in fitting parameters with temperature shown in figure S4 to higher temperatures. (a) shows the differential FTIR spectra of absorbance features across a range of temperatures (350–550 K) compared to room temperature for TM-TES calculated from the fitted models described by equations S1 and S2. The energy differences (thermal redshift) between the positive and negative peaks are shown in the legend, in meV. (b) shows how the thermal redshift increases linearly with temperature. (c) shows changes in calculated differential spectra for TES-DPA at higher temperatures (350–600 K) based on extrapolating the linear trends calculated in figure S4 above the phase transition at 210 K, with the thermal redshifts at each temperature shown in the legend. The increase in thermal redshift due to temperature is shown in (d).

Degenerate and Nondegenerate IR pump–IR probe spectroscopy of Anth-Napth, TM-TES, and TES-DPA

To measure heat energy redistribution in three high-mobility organic semiconductor samples (anth-napth, TM-TES and TES-DPA), degenerate and nondegenerate IR pump–IR probe was employed. IR detector calibration was performed using a standard with known FTIR spectrum for each range (TM-TES at 2000–2250 cm^{-1} and polystyrene at 2850–3000 cm^{-1}), for which wavenumber values could be mapped linearly to detector pixels using least-squares regression. Figure S6 shows the results of degenerate transient absorption spectroscopy focused on the alkyl stretching modes at 2800–3100 cm^{-1} in these samples. Despite the presence of two prominent absorption features in this range in anthnapth, no transient signal was detected in this sample, shown in figure S6a,b. However, some interference is seen when the pump and probe are overlapped in time (for $T_w < 1$ ps). The lack of transient signal in the C–H stretching mode region of anth-napth may be due to

insufficient pump power in this range or simply due to increased scatter effects at higher IR frequencies. The figure also presents 2D plots of the degenerate transient signal, showing the aforementioned interference around time zero in the case of anth-napth. In TM-TES and TES-DPA, the shortlived transient features with decay times of <3 ps described in the work can be seen. In both samples, these signals seem to persist weakly until at least 25 ps after their initial decay, which could suggest some competing process limiting decay of these excited states. At negative probe delay times, when the probe arrives before the pump, perturbed free induction decay (PFID) is observed, although it is difficult to distinguish contributions from each of the five overlapping modes in the pumped spectral region. PFID is seen when the probe pulse induces a coherent polarisation, which emits within the pumped wavenumber range as it decays. Modelling the PFID may reveal otherwise unobserved transitions in this range(5).

In the case of anth-napth, no IR-active feature was found corresponding to the alkyne stretching mode, and so this sample was not appropriate for non-degenerate transient absorption experiment. Figure S7 shows 2D plots representing the time dependence of the nondegenerate transient absorption spectra of TM-TES and TES-DPA. Similarly to the degenerate case, PFID is seen where the probe arrives before the pump. The time dependence of the fitted positive and negative peaks in the transient spectra corresponding to the hot ground state of the alkyne stretching modes are also shown, which show the redshift of the differential spectrum as the sample heats up due to the IR pump laser.

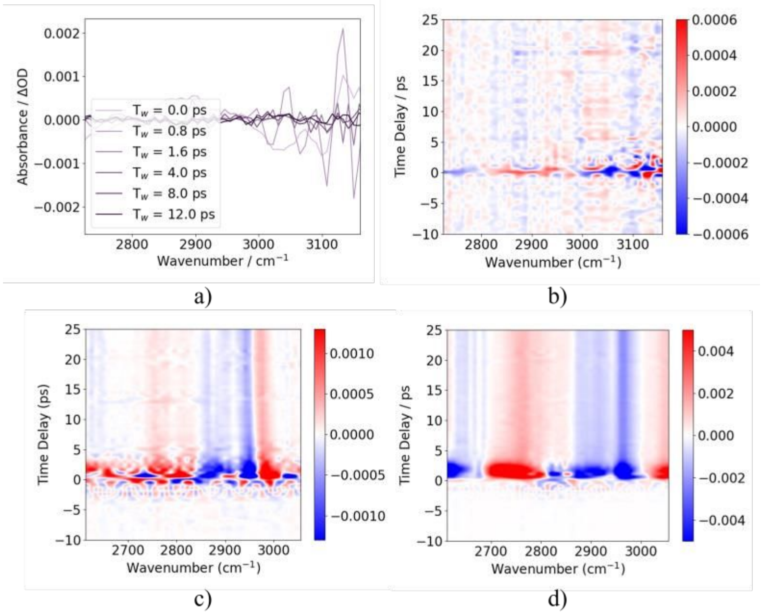

Figure S6) (a) Degenerate (2900 cm^{-1} pump–2900 cm^{-1} probe) transient vibrational absorption spectra for anth-napth, up to a probe delay of 12 ps. (b–d) show 2D representations of the degenerate IR pump–IR probe experiment around time zero, where the pump and probe are overlapped. (b) anth-napth, corresponding to figure S6a, (c) TM-TES, corresponding to figure 3a of the work and (d) TES-DPA, corresponding to figure 3b of the work.

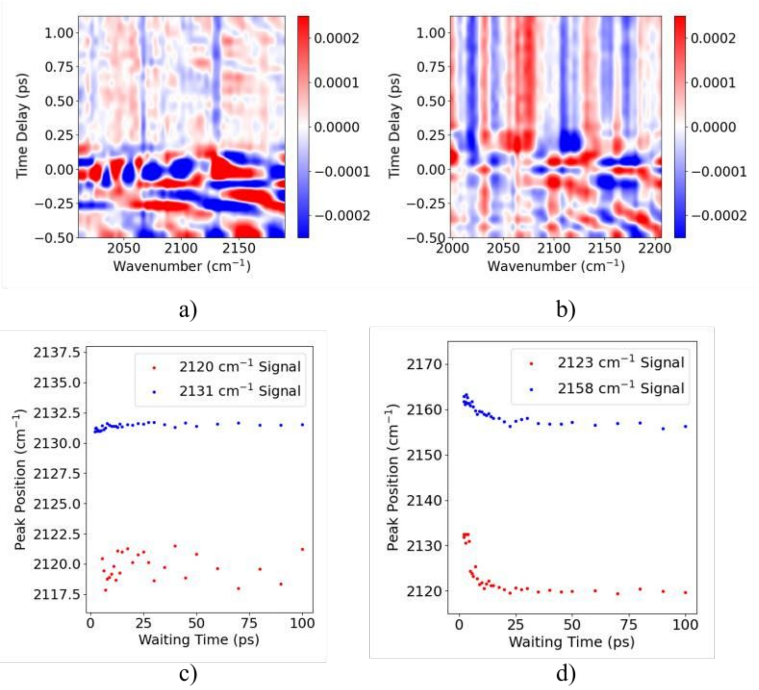

Figure S7) 2D representations of the time dynamics nondegenerate (2900 cm^{-1} pump–2130 cm^{-1} probe) transient vibrational absorption spectra in the probe delay range from -0.5 ps to 1.1 ps for (a) TM-TES and (b) TES-DPA. (c) shows the time dynamics of the peak positions of the fitted positive and negative transient peaks at 2130 cm^{-1} in TM-TES in the probe delay range from -5 to 25 ps and (d) shows the time dynamics of the fitted positive and negative transient peaks at 2130 cm^{-1} in TES-DPA in the probe delay range from -5 to 25 ps.

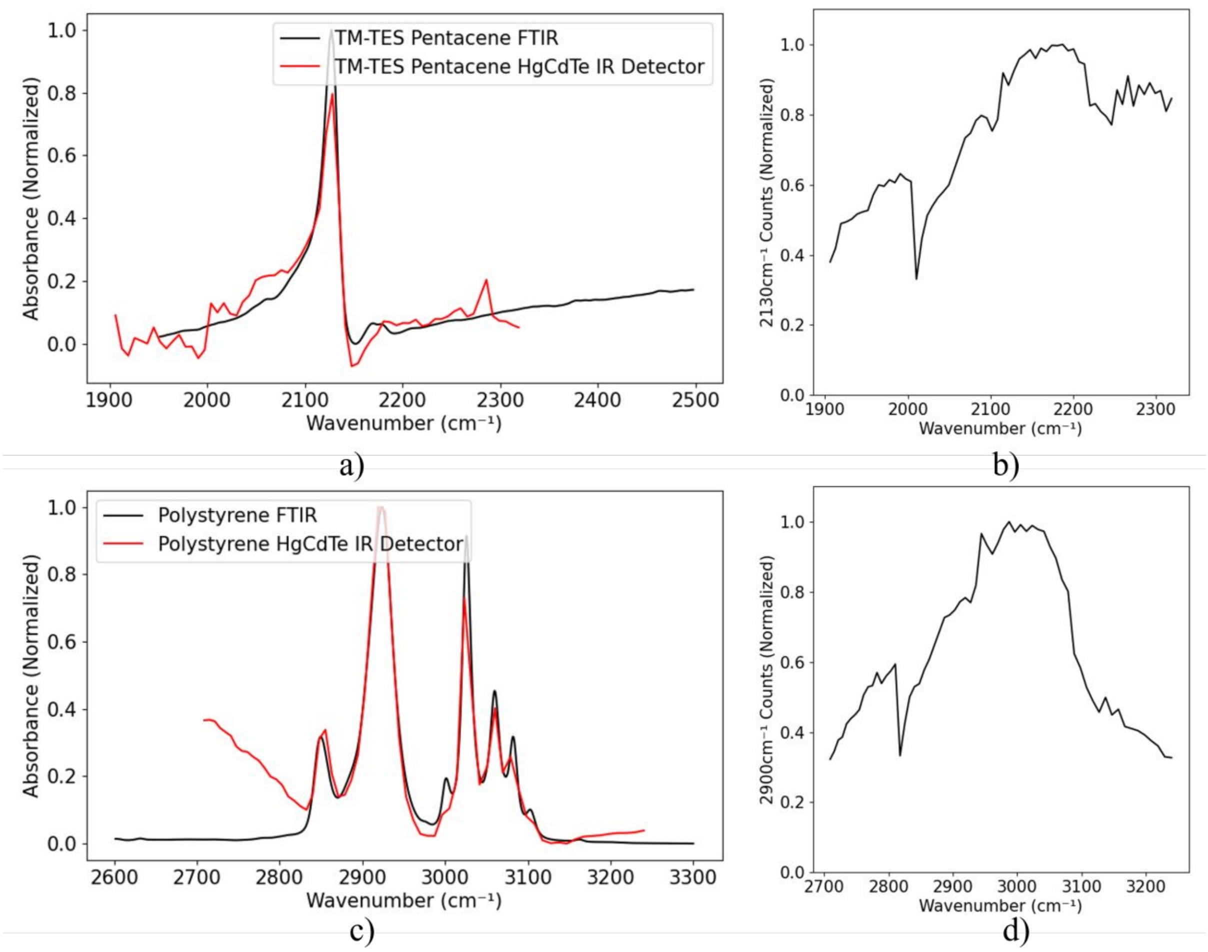

Figure S8) a) Calibration spectra and b) probe spectrum around 2100cm^{-1}. c) Calibration spectra and d) pump spectrum around 2950cm^{-1}.

References

1. Ibrahim MM, MacIel AC, Watson CP, Madec MB, Yeates SG, Taylor DM. Thermo-mechanical stabilisation of a crystalline organic semiconductor for robust large area electronics. Org Electron. 2010;11(7):1234–41. doi:10.1016/j.orgel.2010.04.030

2. Asher M, Jouclas R, Bardini M, Diskin-Posner Y, Kahn N, Korobko R, et al. Chemical Modifications Suppress Anharmonic Effects in the Lattice Dynamics of Organic Semiconductors. ACS Materials Au. 2022 Nov 9;2(6):699–708. doi:10.1021/acsmaterialsau.2c00020

3. Cho M. Molecular photothermal effects on time-resolved IR spectroscopy. Journal of Chemical Physics. 2022 Sep 28;157(12). doi:10.1063/5.0108826 PubMed PMID: 36182430.

4. Balkanski M, Wallis RF, Haro E. Anharmonic effects in light scattering due to optical phonons in silicon. PHYSICAL REVIEW. 1983. Report.

5. Keat TJ, Coxon DJL, Cruddace RJ, Stavros VG, Newton ME, Lloyd-Hughes J. The 3237 cm−1 diamond defect: Ultrafast vibrational dynamics, concentration calibration, and relationship to the N3VH0 defect. Diam Relat Mater. 2024 Jan 1;141. doi:10.1016/j.diamond.2023.110661
